# Effects of Pregnancy Anesthesia on Fetal Nervous System

**DOI:** 10.3389/fphar.2020.523514

**Published:** 2021-02-01

**Authors:** Xingyue Li, Xi Jiang, Ping Zhao

**Affiliations:** ^1^ Department of Anesthesiology, Shengjing Hospital of China Medical University, Shenyang, China; ^2^ Department of Neurosurgery, Shenyang Chest Hospital, Shenyang, China

**Keywords:** maternal anesthesia, mid-trimester, anesthetics, developmental neurotoxicity, cognitive dysfunction

## Abstract

The effects of general anesthesia on the developing brain remain a great concern in the medical field and even in the public, and most researches in this area focus on infancy and childhood. In recent years, with the continuous development of medical technology, the number of operations during pregnancy is increasing, however, studies on general anesthesia during pregnancy are relatively lacking. The mid-trimester of pregnancy is a critical period, and is regarded as a safe period for surgery, but it is a fragile period for the development of the central nervous system and is particularly sensitive to the impact of the environment. Our research group found that general anesthesia may have adverse effects on fetal neurodevelopment during the mid-trimester. Therefore, in this review, we summarize the characteristics of anesthesia during pregnancy, and the related research of the anesthesia’s impacts on the development of central nervous system were introduced.

## Introduction

With the development of medical technology, the number of fetus and infant surgery have been increasing. In the early stage of life whether exposure to general anesthetics impacts the long-term central nervous system adversely has become a global concern for medical professionals and the public. After years of researches and observations, animal studies have confirmed that the application of anesthetics would cause neurodegenerative changes and abnormal learning abilities in brain development. Since 2017, the United States food and drug administration (FDA) has issued a warning about anesthetics and the developing brain—where the neurotoxicity of anesthetics has captured the more attention of the public.

Advances in medicine have made it possible to perform more operations in the fetuses or on newborn babies, This means that during the development stage of the brain, the rate of fetal anesthesia exposure rises. Although the application of sedation or general anesthesia is usually considered safe, recent studies in animals and children have evoked a problem: general anesthesia may lead to adverse effects on neurodevelopment. The main content of the FDA warning is that in late pregnancy or in children less than 3 years old, repeated or long time (more than 3 h) use of general anesthetics or sedatives may affect neurodevelopment. However, for other stages of brain development, the conclusion is not yet explicit. The neurotoxicity of anesthetics is influenced by drug toxicity, dosage, duration of anesthetic exposure, and the embryonic brain development stage (Cheek and Baird, 2009). Genetic predisposition may play a role in it as well. This review introduces the characteristics of anesthetics, the particularity of anesthesia during pregnancy and the effects of anesthetics on the central nervous system during the mid-trimester. Moreover, the suggestions and prospects of maternal anesthesia are proposed.

### Characteristics of Anesthesia During Pregnancy

Particular attention should be paid to the characteristics of the implementation of general anesthesia in a special population such as parturient individuals. Different from common general anesthesia, the placental barrier plays an important role in the transfer and distribution of the drugs in mothers and babies. Due to technical limitations, information on the metabolic and pharmacodynamic of anesthetics in the fetus is limited. Since most anesthetics are fat-soluble (Zheng et al., 2013), it can easily to cross the placenta (De Tina and Palanisamy, 2017), and the effect of placental barrier on drug transport is therefore studied first. Li found that after 6 h of 1.3% isoflurane anesthesia, isoflurane concentrations in the fetal brain and maternal brain were considerable (0.40 and 0.42 mmol/g) (Li et al., 2007). In another study, however, the concentration at the end of fetal respiration was 0.45% below halothane anesthesia with a minimum alveolar concentration (MAC) (Gregory et al., 1983). Similar to inhaled anesthetics, propofol also easily passes through the placenta, with a higher concentration ratio of plasma in the mother/fetus (5.4 and 0.35 mg/ml) (Ngamprasertwong et al., 2016). However, the maternal application of muscle relaxants has little or no effect on the fetus, because of their limited placental penetration (Kampe et al., 2003). These results show that the anesthetic transshipment in the maternal and fetus body is a complex pharmacokinetic model. In clinical practice, it was found that general anesthetics caused an abnormal fetal heart rate after passing through the placenta. To study the effect of the anesthetic on the fetus directly, transport and metabolics of the anesthetic in the fetus and mother should be clear.

Due to the particularity of pregnancy, the MAC value of inhaled anesthetics is reduced (Rosen, 1999), and the sensitivity of drugs is also increased (Zhan et al., 2011). In addition to emergency cesarean section surgery, most fetuses are "bystanders", so there is little requirement of anesthesia in the fetus itself (Ngamprasertwong et al., 2013), for example, MAC of halothane in fetal sheep is the mother's at 50% (0.33% and 0.69%) (Gregory et al., 1983). During pregnancy operation, anesthetic concentration also needs to ensure that the usage is suitable for the anesthesia depth and reduction in the risk of fetal preterm labor, and high concentrations of inhalation anesthetics (about 1.5 MAC) also have the effect of cervical relaxation (De Tina and Palanisamy, 2017). Therefore, the concentration of drugs during pregnancy is also an important factor in anesthesia. Furthermore, in the treatment of diaphragmatic hernia or a meningomyelocele cyst, diseases that usually require surgery and anesthesia (Andropoulos, 2018), the FDA warning content also suggests that repeated anesthesia is one of the key factors that leads to anesthetic neurotoxicity.

### Development of the Fetal Nervous System

The formation of the human nervous system is a continuous, accurate and orderly process, including neural stem cell (NSC) proliferation, differentiation, neuronal migration, and synaptic formation—the process where neurons are precisely positioned to establish synaptic connections with other cells at specific locations (Sanes et al., 2006). According to pregnancy and during the postnatal period, the main content of the development of the nervous system is different, and the level of brain development is not exactly the same in humans and animals. For example, synaptic formation, which is of great concern, occurs mainly during late pregnancy and lasts for 2–3 years after birth in humans (Dobbing and Sands, 1979), but in rodents, it begins to form postnatally.

The main content of the development of the nervous system during mid-trimester is worth noting. Prior to synaptic formation, the main content of neurodevelopment is neurogenesis and neuronal migration, and in both humans and rodents it starts and reaches the peak mid-trimester. Neurogenesis refers to neural stem cells/progenitor cells from the subventricular (SVZ) and subgranular (SGZ) that produce mature neurons through proliferation and division (Sanes et al., 2006). In the developing brain, neural stem cells proliferate a large number of neurons every minute, and these neurons, integrate with other neurons to form neural circuits through migration (Avet-Rochex et al., 2014). The neurodevelopmental processes of this period, neurogenesis and neuronal migration, are extremely sensitive to the environment and drug effects. Therefore, the regulation of neurogenesis is crucial for the formation of a functional nervous system, especially the balance of cell proliferation, differentiation, and survival. Late pregnancy is a period during which the prefrontal cortex (PFC) development takes place. It plays a key role in the development of advanced cognitive function and pathogenesis of many neurodevelopmental deficits (Huang and Vasung, 2014). In late pregnancy and during the infant period, nerve cell apoptosis is a natural physiological process—every day 1% of the nerve cells are affected (Istaphanous et al., 2013).

### Clinical Study and Animal Study

Although a variety of animal studies have shown that general anesthesia may have adverse effects on the developing brain, there is no study on the effects of anesthesia exposure on the neurodevelopment of human fetuses (Andropoulos, 2018). Yet no anesthetics have been explicitly defined as a human teratogen. An international database of fetal anesthesia (Clinical Trials.gov identifier NCT02591745) was established to study the long-term neurodevelopmental outcomes of individuals after the intervention during pregnancy (Olutoye et al., 2018a). Although randomized controlled studies remain the gold standard, due to ethical and technical restrictions, the effects of anesthetics on fetuses can at present, only be studied with relevant animal models. According to translational relevance, non-human primates are the most suitable animal models, but there are few related studies. The motivations for basic research are to identify specific molecular targets and to elucidate the molecular, cellular, and systemic mechanisms of anesthetic neurotoxicity. Based on this, a clinical study could in turn verify and support focused experimental theory (Disma et al., 2018). The existing clinical study showed that women undergoing surgery and anesthesia during pregnancy delivered earlier and preterm more frequently (Devroe et al., 2019). As a retrospective study, its ability to correct confounding variables is limited and is prone to bias. There was another study demonstrating that general anesthesia was associated with a higher number of low birth weight neonates (Jenkins et al., 2003). A study which evaluated gravidas since 2017 found that pregnant women who experienced non-obstetric interventions were more likely to have a poor prognosis (Balinskaite et al., 2017). The disorder in pregnancy may cause developmental abnormalities or premature birth, and it is not clear how much of a role anesthesia plays in that. Thus, these results may be due to disease rather than anesthesia, and no accurate results have been reported on the neurodevelopment consequence yet.

### Mid-Trimester and Anesthesia

In recent years, with the continuous development of diagnosis and surgical technologies, fetal surgery is increasing. Mid-pregnancy is also regarded as a relatively safe period for surgery: avoiding the vulnerable period of fetal organogenesis and high risks of premature delivery (De Tina and Palanisamy, 2017). Under controlled and predictable conditions, using inhalation anesthetics has become a preferred method in uterine surgery to meet the needs of open fetal surgery (Olutoye et al., 2018a). In the past, high concentrations of inhalation anesthetics were usually used to ensure adequate uterine relaxation, optimum exposure to fetal surgery, and to minimize the risk of placental separation (Myers et al., 2002; Garcia et al., 2011). However, people gradually realized that a relatively safe operation period is not necessarily safe for the development of the fetal nervous system, because the second trimester is a critical period of accelerated neurogenesis and neural migration, which is vulnerable to interference from external environmental factors, and subtle changes may lead to long-term nervous system abnormalities. Therefore, the fetal brain in the second trimester may be more susceptible to the adverse effects of inhalation anesthetics.

Because the important events of fetal rodents brain development at the embryonic stage of 14–16 days correspond to those in human mid-trimester, the results of rodent studies at the second trimester are of a certain reference value (Clancy et al., 2001; Clancy et al., 2007). According to an article in *Anesthesiology*, a single exposure to 1.4% isoflurane (1MAC) for 4 h in the second trimester (14 days of gestation) caused long-term spatial working memory impairment, even personality and emotional disorders in adolescence, and alleviated anxiety in offspring rats (Palanisamy et al., 2011). Since then, people have realized that anesthesia during the mid-trimester may have an impact on the nervous system and cognitive function, and extensive researches have therefore been conducted. These studies suggested that general anesthesia in pregnant animals may induce neuroinflammation, caspase activation and synaptic loss, and even lead to learning and memory impairment. Moreover, our group found that the long-term learning and behavioral abnormalities of offspring caused by general anesthesia during the second trimester were due to the influence of apoptosis and proliferation of NSCs (Wang et al., 2018a; Wang et al., 2018b). Next, we introduce the relevant research results according to the duration, frequency, and depth of anesthesia.

### Duration of Anesthesia

The duration of anesthesia is an important factor, as the length of anesthesia causes different effects. A brief (1 h) exposure at 1 MAC or 1.5 MAC enhanced the proliferation of NSCs possibly via ERK1/2, whereas 6 h of exposure reduced the self-renewal capacity, such as inhibited proliferation and increased apoptosis (Nie et al., 2013). Similarly, 6 h 2.5% maternal sevoflurane exposure led to cell cycle arrest of fetal prefrontal nerve precursor cells, resulting in decreased neuron output and inhibited neuron precursor cell replication, which ultimately led to learning deficits in offspring (Song et al., 2017). Since the exposure time (4–6 h) in rodent gestation is the equivalent of 48 h anesthesia in a pregnant woman (Palanisamy, 2012), we judged that long-term duration in the animal studies falls short of the clinical guidance value. However, a shorter duration of anesthesia may not be particularly significant for related studies, as a longer duration of anesthesia is a more recognized adverse factor. Moreover, if the anesthesia time is too short in the animal experiment, it may be easy to cause an inaccurate anesthesia duration due to an improper experimental process, resulting in a large deviation, thus affecting the results. Choosing the appropriate duration of anesthesia in the study is therefore essential.

### Frequency of Anesthesia

Additionally, multiple anesthesia is another possible risk factor. Clinically, fetuses are often subjected to repeated anesthesia and surgery to treat diseases such as myelocele. In a study, exposure to sevoflurane single or multiple times during the second trimester in pregnant rodents did not lead to long-term behavioral consequences or changes in synaptic plasticity in their offsprings (Lee et al., 2017). However, other studies observed detrimental effects of multiple maternal exposures to sevoflurane on the fetal brain. The underlying mechanisms involve the down-regulated cell-cycle factor Ccnd1 by inhibiting Pax6 expression, thereby damaging the neurogenesis of fetal brain and leading to cognitive dysfunction in offspring (Fang et al., 2017). Similarly, our research results showed that a single sevoflurane exposure did not affect the learning and memory abilities of the offspring rats, and repeated sevoflurane exposure resulted in learning and memory impairment in offspring rats (Wu et al., 2018). The reason is that repeated, but not single, exposure to sevoflurane caused premature NSC differentiation, decreased histone acetylation and BDNF expression in fetal brain tissues and the postnatal hippocampus, with the decrease of dendritic spines, then impaired spatially dependent learning and memory functions (Wu et al., 2018; Zhang et al., 2020). In addition to rodent studies, single anesthesia exposure did not show significant neuronal apoptosis in a mid-pregnancy fetal sheep study using clinically relevant doses and exposure times of isoflurane, but neuronal damage was observed after repeated anesthesia exposure (Olutoye et al., 2016). These studies further indicated that the frequency of anesthesia exposure should be considered in the critical neurogenesis stage of brain development.

### Concentration of Anesthetic

Given the FDA warning, duration and frequency are considered key factors in anesthesia-induced neurotoxicity. Relevant studies show that the dose of the anesthetics may also play an important role. However, so far, there is no authoritative safe concentration range. As a clinician, it is more valuable to understand the effects of anesthetic concentration, since the frequency and duration of anesthesia are difficult to control. In an earlier study, exposure to high concentrations of isoflurane (3%) during pregnancy resulted in spatial memory and learning disorders and more neurodegenerative changes in offspring rats compared to the control group or 1.3% isoflurane group. The 3% isoflurane treatment group showed an increased number and optical density of caspase-3 neurons and remarkable changes in synaptic ultrastructure (Kong et al., 2012).

We were interested in whether sevoflurane, which is more commonly used clinically and is less toxic, also has concentration-dependent neurotoxicity. Our data demonstrated that sevoflurane exposure during the mid-trimester caused NSC apoptosis and impairs postnatal learning and memory function in a dose-dependent manner (Wang et al., 2018a). However, the mid-trimester is a critical time for NSC proliferation. If NSC self-renewal can compensate for cell death, fetal neurodevelopment may not be affected. Therefore, apoptosis is not the only mechanism, and there may be an imbalance between cell proliferation and death. The findings of our study suggest that fetal NSC proliferation is inhibited by 3.5% sevoflurane maternal exposure during the mid-trimester (Wang et al., 2018a). In past studies, relevant researches mainly expounded on the mechanism of apoptosis, such as mitochondrial injury, intracellular calcium imbalance, neuroinflammatory (Lei et al., 2012). In addition to apoptosis and proliferation, we found that sevoflurane anesthesia during the mid-trimester upregulated autophagy levels of the NSCs concentration-dependently. We also demonstrat that autophagy is related to the apoptosis increase and proliferation inhibition induced by sevoflurane. Autophagy inhibition alleviated NSC apoptosis, proliferation inhibition and learning and memory impairment (Li et al., 2017). Since then, several studies have reported the correlation between anesthetics and autophagy. The high concentration of propofol had adverse effects on the neural precursor cell viability *in vitro* through Ca^2+^ mediated pathway of over-activated autophagy (Qiao et al., 2017). Ketamine-induced neurotoxicity was caused by ROS-mediated activation of mitochondrial apoptotic pathway and autophagy (Li et al., 2018).

In particular developmental stages during pregnancy, any significant high-dose, prolonged duration of drugs can theoretically lead to teratogenicity, but not all anesthetics showed detrimental effects. Similar to the results above, isoflurane exposure in mid-trimester mice led to embryo neuroinflammation, apoptosis, and impaired cognitive function, however, a combination of propofol alleviated these adverse effects (Nie et al., 2020). Besides, in one of the mid-trimester studies, high doses of dexmedetomidine for 12 h at 120 days of gestation (mid-pregnancy) did not affect neuronal apoptosis in the frontal cortex of the fetal cynomolgus monkey brain (Koo et al., 2014). The combined use of dexmedetomidine and isoflurane during the second trimester of pregnancy (14 days of pregnancy) can alleviate the impairment of spatial learning and memory function (Su et al., 2015). Besides anesthesia, surgical stimulation appears to reduce anesthesia-induced neuroapoptosis in the mid-trimester ovine fetus (Olutoye et al., 2018b). Further studies are needed to determine the long-term effects and consequences of these findings on fetal and uterine anesthesia and postoperative behavioral and cognitive functions. These studies may provide a wealth of information to prevent or treat the associated adverse effects of anesthesia during the mid-trimester.

### Pain Management

While animal studies mimic anesthesia, they ignore the effects of surgical pain on the fetus. Before 22 weeks, the fetus has no neuroanatomical pathway to feel pain; between 22 and 26 weeks, thalamocortical fibers, which are crucial for pain perception, start forming; after 26 weeks, the fetus develops the necessary nervous system to feel pain (Littleford, 2004). The effects of pain on neurodevelopment are however unclear. Liu found that pain during emergency operations and procedures can reduce neuroapoptosis caused by ketamine (Liu et al., 2012). Appropriate pain management is also important, because improper pain management may lead to a premature delivery. If there are no contraindications, acute pain can be treated with an epidural block and peripheral nerve blocks (Okeagu et al., 2020).

### Consensus and Clinical Advice

Based on current knowledge of anesthesia and surgery during pregnancy, intervention is risky for both the mother and the fetus. First, elective surgery should be avoided during pregnancy and be delayed as far as possible until delivery (Flood, 2011). Regional anesthesia has always been preferred for the operations that must be conducted during pregnancy. Regional anesthesia has the advantages of effectively reducing pain and having little impact on fetal heart tone variability (Cheek and Baird, 2009) highlight, but not all surgical operations can be carried out under regional anesthesia. Secondly, although spinal anesthesia is still the main anesthesia management for patients during pregnancy, general anesthesia, mainly inhalation anesthesia, is also a common method of emergency non-obstetric or open fetal surgery. Unfortunately, the FDA warning does not provide recommended concentrations and dose-related guidelines for anesthetics avoid neurodevelopment damage. Since there is a lack of relevant guidelines, we suggest to minimize the exposure duration and avoid high concentration anesthetics, for instance by limiting the interval from anesthesia induction to operation start to reduce patients' anesthetic exposure. It is important and appropriate that the fetus should be exposed to anesthetics for as short as possible.

The health of fetuses and newborns is the main criterion for evaluating obstetric operations and anesthesia management of pregnant women, but not all infants can benefit from early intervention or fetal surgery, and more work is needed to determine this. In the future, we need to explore techniques to predict the outcome of pregnancy surgery by measuring the basic characteristics of the fetus, and to develop fetal intervention methods that cause less trauma. At the same time, intraoperative fetal monitoring (Doppler ultrasound and uterine contraction monitoring) is also significantly important (Okeagu et al., 2020). Further studies are needed to determine the best anesthesia method to ensure maternal and fetal cardiovascular stability, optimal uterine perfusion, adequate uterine relaxation, sufficient anesthesia depth, minimal fetal myocardial inhibition, and appropriate blocking of the fetal stress response (Sviggum and Kodali, 2013). During the observation period, attention should also be paid to keeping the umbilical connection between the fetus and mother to avoid affecting the neurological, biochemical and behavioral functions of the fetus due to the decrease of oxygen and nutrient supply (Ronca et al.,2007). Finally, proper communication and teamwork between anesthesiologists, obstetricians, and pediatricians is necessary to ensure the safety of pregnant women and fetuses.

### Expectation

With the rapid advancement of medical technology, anesthesiologists are facing a challenge in pregnancy anesthesia: how to balance the needs of mothers and fetuses/newborns, and how to adjust methods flexibly in specific circumstances. Ideal pregnant anesthetics or techniques should be safe and effective for mothers, without harming the fetus in the uterus or affecting the normal labor process, providing satisfactory delivery conditions, allowing early interaction between mothers and infants, without short-term or long-term effects on newborns (Littleford, 2004), For example, studies have shown that dexmedetomidine significantly reduced spatial learning and memory impairment, but its neuroprotective mechanism for fetuses remains to be studied further (Koo et al., 2014). In addition to dexmedetomidine, protective or mitigating strategies are under consideration, including cytoskeletal stabilizers jasplakinolide or TAT-Pep5, antioxidants resveratrol, vitamin C, L-carnitine, arachidonic acid, β-estradiol, lithium. However, these agents cannot be recommended as protective or mitigating strategies at present, and more rigorous research is needed. In the future, based on more in-depth animal experiments, we should carry out multicenter randomized controlled tests to obtain authoritative experimental conclusions as soon as possible.

In short, the emergence of new anesthetics, the improvement of anesthesia technology, and the deep understanding of the placental transport and mechanisms of drug action are all of great significance to the prognosis of mothers and infants. The anesthetic neurotoxicity could be a problem in anesthesia practice. However, family, life events and social environment may also be the major determinants of neurodevelopment and long-term cognitive functions (Koo et al., 2014). In the future, continuous efforts and explorations by anesthesiologists are recommended.

## Author Contributions

XL wrote the review with the help of XJ. PZ revised the article.

## Funding

This work was supported by the National Nature Science Foundation of China (grant number 81870838), Liaoning Province Distinguished Professor Support Program (grant number XLYC1802096), Shenyang Clinical Medicine Research Center of Anesthesiology (grant numbers 19-110-4-24, No. 20-204-4-44), and the Outstanding Scientific Fund of Shengjing Hospital (grant number 201708).

## Conflict of Interest

The authors declare that the research was conducted in the absence of any commercial or financial relationships that could be construed as a potential conflict of interest.

## References

[B1] Andropoulos D. B. (2018). Effect of anesthesia on the developing brain: infant and fetus. Fetal Diagn. Ther. 43 (1), 1–11. 10.1159/000475928 28586779

[B2] Avet-Rochex A. Carvajal N. Christoforou C. P. Yeung K. Maierbrugger K. T. Hobbs C. (2014). Unkempt is negatively regulated by mTOR and uncouples neuronal differentiation from growth control. PLoS Genet. 10 (9), e10j.1460-9592.2002.008424 10.1371/journal.pgen.1004624 PMC4161320 25210733

[B3] Balinskaite V. Bottle A. Sodhi V. Rivers A. Bennett P. R. Brett S. J. (2017). The risk of adverse pregnancy outcomes following nonobstetric surgery during pregnancy: estimates from a retrospective cohort study of 6.5 million pregnancies. Ann. Surg. 266 (2), 260–266. 10.1097/SLA.0000000000001976 27617856

[B4] Cheek T. G. Baird E. (2009). Anesthesia for nonobstetric surgery: maternal and fetal considerations. Clin. Obstet. Gynecol. 52 (4), 535–545. 10.1097/GRF.0b013e3181c11f60 20393407

[B5] Clancy B. Kersh B. Hyde J. Darlington R. B. Anand K. J. Finlay B. L. (2007). Web-based method for translating neurodevelopment from laboratory species to humans. Neuroinformatics 5 (1), 79–94. 10.1385/ni:5:1:79 17426354

[B6] Clancy B. Darlington R. B. Finlay B. L. (2001). Translating developmental time across mammalian species. Neuroscience 105 (1), 7–17. 10.1016/s0306-4522(01)00171-3 11483296

[B7] De Tina A. Palanisamy A. (2017). General anesthesia during the third trimester: any link to neurocognitive outcomes? Anesthesiol. Clin. 35 (1), 69–80. 10.1016/j.anclin.2016.09.007 28131121

[B8] Devroe S. Bleeser T. Van de Velde M. Verbrugge L. De Buck F. Deprest J. (2019). Anesthesia for non-obstetric surgery during pregnancy in a tertiary referral center: a 16-year retrospective, matched case-control, cohort study. Int. J. Obstet. Anesth. 39, 74–81. 10.1016/j.ijoa.2019.01.006 30772120

[B9] Disma N. O'Leary J. D. Loepke A. W. Brambrink A. M. Becke K. Clausen N. G. (2018). Anesthesia and the developing brain: a way forward for laboratory and clinical research. Paediatr. Anaesth. 28 (9), 758–763. 10.1111/pan.13455 30117228

[B10] Dobbing J. Sands J. (1979). Comparative aspects of the brain growth spurt. Early Hum. Dev. 3 (1), 79–83. 10.1016/0378-3782(79)90022-7 118862

[B11] Fang F. Song R. Ling X. Peng M. Xue Z. Cang J. (2017). Multiple sevoflurane anesthesia in pregnant mice inhibits neurogenesis of fetal hippocampus via repressing transcription factor Pax6. Life Sci. 175, 16–22. 10.1016/j.lfs.2017.03.003 28279665

[B12] Flood P. (2011). Fetal anesthesia and brain development. Anesthesiology 114 (3), 479–480. 10.1097/ALN.0b013e318209aa8c 21278569

[B13] Garcia P. J. Olutoye O. O. Ivey R. T. Olutoye O. A. (2011). Case scenario: anesthesia for maternal-fetal surgery: the Ex Utero Intrapartum Therapy (EXIT) procedure. Anesthesiology 114 (6), 1446–1452. 10.1097/ALN.0b013e31821b173e 21555935

[B14] Gregory G. A. Wade J. G. Beihl D. R. Ong B. Y. Sitar D. S. (1983). Fetal anesthetic requirement (MAC) for halothane. Anesth. Analg. 62 (1), 9–14. 6849513

[B15] Huang H. Vasung L. (2014). Gaining insight of fetal brain development with diffusion MRI and histology. Int. J. Dev. Neurosci. 32, 11–22. 10.1016/j.ijdevneu.2013.06.005 23796901 PMC3825830

[B16] Istaphanous G. K. Ward C. G. Nan X. Hughes E. A. McCann J. C. McAuliffe J. J. (2013). Characterization and quantification of isoflurane-induced developmental apoptotic cell death in mouse cerebral cortex. Anesth. Analg. 116 (4), 845–854. 10.1213/ANE.0b013e318281e988 23460572

[B17] Jenkins T. M. Mackey S. F. Benzoni E. M. Tolosa J. E. Sciscione A. C. (2003). Non-obstetric surgery during gestation: risk factors for lower birthweight. Aust. N. Z. J. Obstet. Gynaecol. 43 (1), 27–31. 10.1j.1460-9592.2002.0084/j.0004-8666.2003.00001.x 12755343

[B18] Kampe S. Krombach J. W. Diefenbach C. (2003). Muscle relaxants. Best Pract. Res. Clin. Anaesthesiol. 17 (1), 137–146. 10.4045/tidsskr.08.0323 12751553

[B19] Kong F. J. Ma L. L. Hu W. W. Wang W. N. Lu H. S. Chen S. P. (2012). Fetal exposure to high isoflurane concentration induces postnatal memory and learning deficits in rats. Biochem. Pharmacol. 84 (4), 558–563. 10.1016/j.bcp.2012.06.001 22705347

[B20] Koo E. Oshodi T. Meschter C. Ebrahimnejad A. Dong G. (2014). Neurotoxic effects of dexmedetomidine in fetal cynomolgus monkey brains. J. Toxicol. Sci. 39 (2), 251–262. 10.2131/jts.39.251 24646706

[B21] Lee S. Chung W. Park H. Park H. Yoon S. Park S. (2017). Single and multiple sevoflurane exposures during pregnancy and offspring behavior in mice. Paediatr. Anaesth. 27 (7), 742–751. 10.1111/pan.13139 28497474

[B22] Lei X. Guo Q. Zhang J. (2012). Mechanistic insights into neurotoxicity induced by anesthetics in the developing brain. Int. J. Mol. Sci. 13 (6), 6772–6799. 10.3390/ijms13066772 22837663 PMC3397495

[B23] Li X. Wu Z. Zhang Y. Xu Y. Han G. Zhao P. (2017). Activation of autophagy contributes to sevoflurane-induced neurotoxicity in fetal rats. Front. Mol. Neurosci. 10, 432 10.3389/fnmol.2017.00432 29311820 PMC5744904

[B24] Li Y. Liang G. Wang S. Meng Q. Wang Q. Wei H. (2007). Effects of fetal exposure to isoflurane on postnatal memory and learning in rats. Neuropharmacology 53 (8), 942–950. 10.1016/j.neuropharm.2007.09.005 17959201 PMC2170454

[B25] Li Y. Li X. Zhao J. Li L. Wang Y. Zhang Y. (2018). Midazolam attenuates autophagy and apoptosis caused by ketamine by decreasing reactive oxygen species in the Hippocampus of fetal rats. Neuroscience 388, 460–471. 10.1016/j.neuroscience.2018.03.040 29634998

[B26] Littleford J. (2004). Effects on the fetus and newborn of maternal analgesia and anesthesia: a review. Can. J. Anaesth. 51 (6), 586–609. 10.1007/BF03018403 15197123

[B27] Liu J. R. Liu Q. Li J. Baek C. Han X. H. Athiraman U. (2012). Noxious stimulation attenuates ketamine-induced neuroapoptosis in the developing rat brain. Anesthesiology 117 (1), 64–71. 10.1097/ALN.0b013e31825ae693 22617253

[B28] Myers L. B. Cohen D. Galinkin J. Gaiser R. Kurth C. D. (2002). Anaesthesia for fetal surgery. Paediatr. Anaesth. 12 (7), 569–578. 10.1046/j.1460-9592.2002.00840.x 12358650

[B29] Ngamprasertwong P. Michelfelder E. C. Arbabi S. Choi Y. S. Statile C. Ding L. (2013). Anesthetic techniques for fetal surgery: effects of maternal anesthesia on intraoperative fetal outcomes in a sheep model. Anesthesiology 118 (4), 796–808. 10.1097/ALN.0b013e318283c954 23343650

[B30] Ngamprasertwong P. Dong M. Niu J. Venkatasubramanian R. Vinks A. A. Sadhasivam S. (2016). Propofol pharmacokinetics and estimation of fetal propofol exposure during mid-gestational fetal surgery: a maternal-fetal sheep model. PloS One 11 (1), e0146563 10.1371/journal.pone.0146563 26752560 PMC4713870

[B31] Nie H. Peng Z. Lao N. Dong H. Xiong L. (2013). Effects of sevoflurane on self-renewal capacity and differentiation of cultured neural stem cells. Neurochem. Res. 38 (8), 1758–1767. 10.1007/s11064-013-1074-4 23756731

[B32] Nie Y. Li S. Yan T. Ma Y. Ni C. Wang H. (2020). Propofol attenuates isoflurane-induced neurotoxicity and cognitive impairment in fetal and offspring mice. Anesth. Analg. 131 (5), 1616–1625. 10.1213/ANE.0000000000004955 33079886

[B33] Okeagu C. N. Anandi P. Gennuso S. Hyatali F. Stark C. W. Prabhakar A. (2020). Clinical management of the pregnant patient undergoing non-obstetric surgery: review of guidelines. Best Pract. Res. Clin. Anaesthesiol. 34 (2), 269–281. 10.1016/j.bpa.2020.04.004 32711833

[B34] Olutoye O. A. Sheikh F. Zamora I. J. Yu L. Akinkuotu A. C. Adesina A. M. (2016). Repeated isoflurane exposure and neuroapoptosis in the midgestation fetal sheep brain. Am. J. Obstet. Gynecol. 214 (4), 542 e541–542 e548. 10.1016/j.ajog.2015.10.927 26546852

[B35] Olutoye O. A. Baker B. W. Belfort M. A. Olutoye O. O. (2018a). Food and Drug Administration warning on anesthesia and brain development: implications for obstetric and fetal surgery. Am. J. Obstet. Gynecol. 218 (1), 98–102. 10.1016/j.ajog.2017.08.107 28888583

[B36] Olutoye O. A. Cruz S. M. Akinkuotu A. C. Sheikh F. Zamora I. J. Yu L. (2018b). Fetal surgery decreases anesthesia-induced neuroapoptosis in the mid-gestational fetal ovine brain. Fetal Diagn. Ther. 46, 111–118. 10.1159/000491925 30317244

[B37] Palanisamy A. (2012). Maternal anesthesia and fetal neurodevelopment. Int. J. Obstet. Anesth. 21 (2), 152–162. 10.1016/j.ijoa.2012.01.005 22405978

[B38] Palanisamy A. Baxter M. G. Keel P. K. Xie Z. Crosby G. Culley D. J. (2011). Rats exposed to isoflurane in utero during early gestation are behaviorally abnormal as adults. Anesthesiology 114 (3), 521–528. 10.1097/ALN.0b013e318209aa71 21307768 PMC3071297

[B39] Qiao H. Li Y. Xu Z. Li W. Fu Z. Wang Y. (2017). Propofol affects neurodegeneration and neurogenesis by regulation of autophagy via effects on intracellular calcium homeostasis. Anesthesiology 127 (3), 490–501. 10.1097/ALN.0000000000001730 28614084 PMC5561483

[B40] Ronca A. E. Abel R. A. Alberts J. R. (2007). Maternal anesthesia via isoflurane or ether differentially affects pre-and postnatal behavior in rat offspring. Dev. Psychobiol. 49 (7), 675–684. 10.1002/dev.20242 17943977

[B41] Rosen M. A. (1999). Management of anesthesia for the pregnant surgical patient. Anesthesiology 91 (4), 1159–1163. 10.1097/00000542-199910000-00033 10519516

[B42] Sanes D. H. Reh T. A. Harris W. A. (2006). Development of the nervous system. Amsterdam, Boston: Elsevier.

[B43] Song R. Ling X. Peng M. Xue Z. Cang J. Fang F. (2017). Maternal sevoflurane exposure causes abnormal development of fetal prefrontal cortex and induces cognitive dysfunction in offspring. Stem Cells Int. 2017, 6158468 10.1155/2017/6158468 29098009 PMC5643154

[B44] Su Z. Xu S. Chen T. Chen J. (2015). Dexmedetomidine protects spatial learning and memory ability in rats. J. Renin Angiotensin Aldosterone Syst. 16 (4), 995–1000. 10.1177/1470320314562059 25501308

[B45] Sviggum H. P. Kodali B. S. (2013). Maternal anesthesia for fetal surgery. Clin. Perinatol. 40 (3), 413–427. 10.1016/j.clp.2013.05.012 23972748

[B46] Wang Y. Yin S. W. Zhang N. Zhao P. (2018a). High-concentration sevoflurane exposure in mid-gestation induces apoptosis of neural stem cells in rat offspring. Neural Regen. Res. 13 (9), 1575–1584. 10.4103/1673-5374.237121 30127118 PMC6126114

[B47] Wang Y. Yin S. Xue H. Yang Y. Zhang N. Zhao P. (2018b). Mid-gestational sevoflurane exposure inhibits fetal neural stem cell proliferation and impairs postnatal learning and memory function in a dose-dependent manner. Dev. Biol. 435 (2), 185–197. 10.1016/j.ydbio.2018.01.022 29410165

[B48] Wu Z. Li X. Zhang Y. Tong D. Wang L. Zhao P. (2018). Effects of sevoflurane exposure during mid-pregnancy on learning and memory in offspring rats: beneficial effects of maternal exercise. Front. Cell. Neurosci. 12, 122 10.3389/fncel.2018.00122 29773978 PMC5943573

[B49] Zhan Q. Huang S. Geng G. Xie Y. (2011). Comparison of relative potency of intrathecal bupivacaine for motor block in pregnant versus non-pregnant women. Int. J. Obstet. Anesth. 20 (3), 219–223. 10.1016/j.ijoa.2011.01.001 21616657

[B50] Zhang Y. Wu Z. Li X. Wan Y. Zhang Y. Zhao P. (2020). Maternal sevoflurane exposure affects differentiation of hippocampal neural stem cells by regulating miR-410-3p and ATN1. Stem Cell Res. Ther. 11 (1), 423 10.1186/s13287-020-01936-9 32993796 PMC7523391

[B51] Zheng H. Dong Y. Xu Z. Crosby G. Culley D. J. Zhang Y. (2013). Sevoflurane anesthesia in pregnant mice induces neurotoxicity in fetal and offspring mice. Anesthesiology 118 (3), 516–526. 23314109 10.1097/ALN.0b013e3182834d5d PMC3580035

